# Bats and Rodents Shape Mammalian Retroviral Phylogeny

**DOI:** 10.1038/srep16561

**Published:** 2015-11-09

**Authors:** Jie Cui, Gilda Tachedjian, Lin-Fa Wang

**Affiliations:** 1 Program in Emerging Infectious Diseases, Duke-NUS Graduate Medical School, Singapore, Singapore; 2 Centre for Biomedical Research, Burnet Institute, Melbourne, Victoria, Australia; 3 Department of Microbiology, Monash University, Clayton, Victoria, Australia; 4 Department of Infectious Diseases, Monash University, Melbourne, Victoria, Australia; 5 Department of Microbiology and Immunology at the Peter Doherty Institute for Infection and Immunity, The University of Melbourne, Parkville, Victoria, Australia

## Abstract

Endogenous retroviruses (ERVs) represent past retroviral infections and accordingly can provide an ideal framework to infer virus-host interaction over their evolutionary history. In this study, we target high quality Pol sequences from 7,994 Class I and 8,119 Class II ERVs from 69 mammalian genomes and surprisingly find that retroviruses harbored by bats and rodents combined occupy the major phylogenetic diversity of both classes. By analyzing transmission patterns of 30 well-defined ERV clades, we corroborate the previously published observation that rodents are more competent as originators of mammalian retroviruses and reveal that bats are more capable of receiving retroviruses from non-bat mammalian origins. The powerful retroviral hosting ability of bats is further supported by a detailed analysis revealing that the novel bat gammaretrovirus, *Rhinolophus ferrumequinum* retrovirus, likely originated from tree shrews. Taken together, this study advances our understanding of host-shaped mammalian retroviral evolution in general.

Most emerging and re-emerging viral diseases of humans are zoonoses that originate in wildlife. The viral spillover starts from the exposure of donors to recipients, and intermediate hosts can also facilitate this process. Virus-host switching among non-human species is known to be an early but key step leading to an outbreak^1^. Human immunodeficiency virus (HIV), that causes acquired immunodeficiency syndrome (AIDS), is an important example of viral spillover by host switching from African primates to humans dating back to the early 20th century^2^.

Retroviruses are the most successful infectious pathogens, underscored by their highly extended host spectrum and the presence of millions of remnant copies in various host genomes. Vertebrate genomes are inundated with a large number of endogenous retroviruses (ERVs), representing the remnants of past integration of exogenous retroviruses (XRVs). Phylogenetically, most ERVs can be grouped with their exogenous counterparts into three ERV classes: Class I (gammaretrovirus-like), Class II (betaretrovirus-like) and Class III (spumavirus-like), with Class I and II the most abundant in terms of copy number and genetic diversity^3^,^4^. Mining of a large number of vertebrate genomes has uncovered a remarkable depth in retroviral sequence diversity, with rats considered the neglected facilitators of Class I ERV spread across diverse mammalian hosts^5^. Mining of three bat genomes, the large flying fox and the little brown bat, has revealed that both diversified Class I and II ERVs have likely circulated in bats throughout their evolutionary history and clustered with their extant counterparts from divergent mammalian lineages indicating the possibility of cross-species transmission^6^,^7^,^8^. In addition, a pan-vertebrate comparative phylogenomics study has revealed a deep host-retrovirus co-evolutionary relationship and frequent host-switching of retroviruses among distantly related vertebrates^9^.

In this study, we improved the analysis by mining diverse mammalian genomes including ten bat genomes by extracting long and good quality Pol sequences. We systematically established a comprehensive retrovirus-host relationship that clarifies the roles of hosts in mammalian retroviral diversification.

## Results and Discussion

### ERV Phylogenetic Diversity

By using our *in silico* genomic mining pipeline, 76,099 ERVs that contain Pol sequences were extracted from 69 mammalian genomes (Table S1). ERVs are not usually favored by natural selection, thus most viral remnants embedded in host genomes have been subjected to inactivating mutations, frequent indels or truncation due to genomic rearrangements^10^. For these reasons, we only included sufficiently long ERV sequences in order to obtain robust phylogenetic signals while ERVs with truncated Pol sequences were removed. Specifically, our analysis included Pol (encoding the reverse transcriptase, RT and integrase, IN) amino acid positions 85–971 (≥887 amino acids) for Class I ERVs (using reticuloendotheliosis virus as the reference) and amino acid positions 5–741 (≥737 amino acids) for Class II ERVs (using Jaagsiekte sheep retrovirus as the reference), respectively. After exclusion of non-informative sequences, mostly representing either sequences that were truncated or that did not fall into either the Class I or II ERV categories, the total numbers of Pol sequences were reduced to 16,113 comprising 7,994 Class I and 8,119 Class I ERVs.

**Figure 1:**
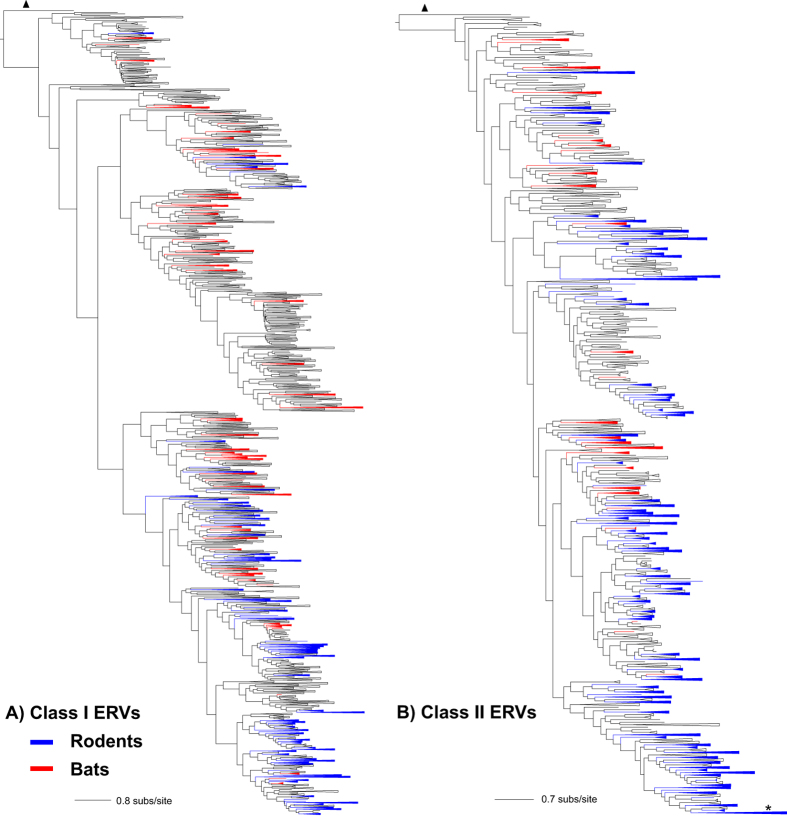
Phylogenetic trees of Class I (**A**) and Class II (**B**) ERVs. Viral branches of rodents are blue highlighted and bats are red. Viruses that formed a same-host clade (*n* ≥ 2) or a similar-host (i.e. from same host family) clade are collapsed into a single lineage for the purpose of visualization only. Branch lengths are drawn to a scale of amino acid substitutions per site (subs/site). The Class I phylogeny is rooted to walleye dermal sarcoma virus, an epsilonretrovirus (marked with a triangle), and the Class II is rooted to avian leukemia virus, an alpharetrovirus (marked with a triangle). The star in the Class II phylogeny represents a primate Class II virus embedded in a rodent clade.

To extract informative phylogenetic signals, ERVs from the same host forming a clade (*n* > 2) with moderate (≥0.70) or high (≥0.90) SH (Shimodaira-Hasegawa) value support were collapsed into a single branch. ERVs from a similar host (i.e. same host family) were also collapsed into the families: Pteropodidae (black flying fox, large flying fox and straw-coloured fruit bat); Vespertilionidae (little brown bat, Brandt’s bat, David’s myotis, and big brown bat); Hominidae and Cercopithecidae (human, chimpanzee, orangutan, and macaque); Cricetidae (golden hamster and Chinese hamster), and Muridae (mouse and rat). After collapse, a pattern of ERVs from bats and rodents was revealed that overwhelmingly occupied most phylogenetic positions (Fig. 1). In the rooted Class I phylogeny (Fig. 1A), bats (order Chiroptera, *n* = 10) tend to harbor more ancient ERVs, while rodents (Rodentia, *n* = 12) have more recently infiltrated retroviruses. Our observation regarding rodent ERVs is in agreement with the more recent endogenization of mice with gammaretroviruses^11^. Notably, although bats have lower total copy numbers of both Class I and II ERVs in their genomes compared to rodents (~2 X and ~14 X lower, respectively) (Table S1), these bat ERVs exhibit comparable, if not greater, phylogenetic diversity. This pattern was demonstrated by calculating the total phylogenetic positions occupied by one species divided by its viral copy number in a particular host species (Fig. S1), an indicator of the ability of bats harboring diverse ERVs. Thus bats tend to harbour a lower number of retroviruses but these viruses tend to occupy more diversified phylogenetic positions, which is unexpected since diversity is normally positively correlated with viral copies as shown in some rodent ERVs^5^.

To compare the ERV pattern of bats and rodents to other host groups, phylogenetic diversities of ERVs found in four additional mammalian orders, such as even-toed ungulates (Artiodactyla, *n* = 6), carnivorans (Carnivora, *n* = 8), cetaceans (Cetacea, *n* = 4), and primates (Primates, *n* = 5), were determined (Fig. S2). Although the carnivorans exhibited the greatest class I ERV diversity compared to the even-toed ungulates, cetaceans and primates (Fig. S2B), none of these four groups harbored the same level of diversity as observed for Class I or II ERVs in bats and rodents (Fig. 1). Some of the hosts among the four groups have considerable viral copies relative to the average viral copy number among all host groups but low phylogenetic diversity, such as even-toed ungulates of Class I ERVs (Fig. S2A) and primates of Class II (Fig. S2H). Phylogenetic analyses revealed that the low phylogenetic diversity of viruses in these hosts such as cetaceans (Fig. S2G) and primates (Fig. S2H) can be explained by frequent retroviral integration into the same host within a short period of time resulting in large numbers of genetically similar retroviruses being densely grouped.

### Cross-species Retroviral Transmission

**Figure 2:**
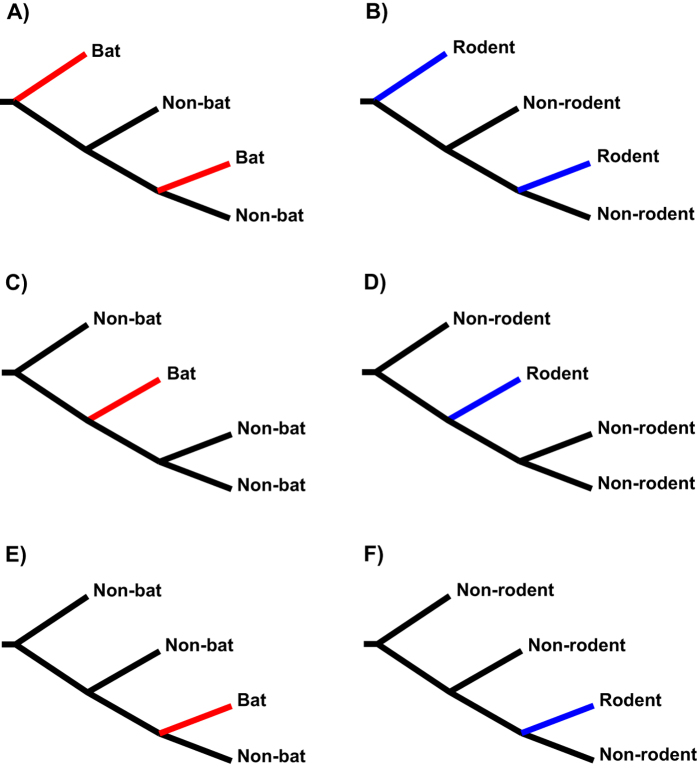
The simplified cross-species transmission patterns of mammalian retroviruses. All the tree topologies are rooted and represent the following patterns: **(A)** originated-from-bat; **(B)** originated-from-rodent; **(C)** transmitted-via-bat; **(D)** transmitted-via-rodent; **(E)** received-by-bat; and **(F)** received-by-rodent.

To determine general cross-species retroviral transmission patterns, 16 clades of Class I (Fig. S3) and 14 of Class II ERVs (Fig. S4) were analyzed. For each clade chosen in this study, each major node (i.e. divergence point of two viruses from different hosts) is supported by high SH values (>0.90) that most likely represents a single introduction of the viruses in different hosts. The majority of the clades are either rodent-specific or bat-specific, indicating limited cross-species transmission between those two host groups, although there are a few (*n* = 3) that contains both rodent and bat ERVs. A total of six cross-species transmission patterns are revealed (Figs S3 and S4): 1) originated-from-bat, where bat branches are positioned at the basal and the internal or end of the phylogeny (Fig. 2A) (clades 1.2, 1.7, 1.11) [note that the topology of (bat,(non-bat,non-bat)) is not considered to belong to this pattern due to the parallel relationship of bat and two non-bats; however the (bat,(bat,non-bat)) topology (infrequently observed) was considered to be most likely originated from bats and hence was included]; 2) originated-from-rodent (Fig. 2B) (clades 1.9, 1.12, 1.13, 1.14, 1.16, 2.3, 2.4, 2.5, 2.6, 2.7, 2.8, 2.10, 2.12, 2.13); 3) transmitted-via-bat, where bat branch (or branches) are positioned internally but not at the end (Fig. 2C) (clades 1.1, 1.3, 1.6); 4) transmitted-via-rodent (Fig. 2D) (clades 1.15, 2.11, 2.14); 5) received-by-bat, where bat branch is located at the end (Fig. 2E) (clades 1.5, 1.8, 1.10, 2.1, 2.2, 2.9, 2.11); and 6) received-by-rodent (Fig. 2F) (clade 1.4). Among the six patterns, the originated-from-rodent (14 cases) is the most dominant, which agrees with the previous finding that rats could serve as major facilitators of Class I ERVs spread across diverse mammalian hosts^5^. Moreover, our data demonstrate that rodents in general are important reservoirs of both Class I and II ERVs. This is especially true for Class II ERVs as 9 of 14 clades are all from rodents. Another interesting finding is the large number (seven cases) of the received-by-bat pattern, in comparison to the one case of received-by-rodent, which seems to be consistent with the observation that bats are ideal reservoir hosts of numerous emerging or re-emerging viruses^12^.

### RfRV Origin

**Figure 3:**
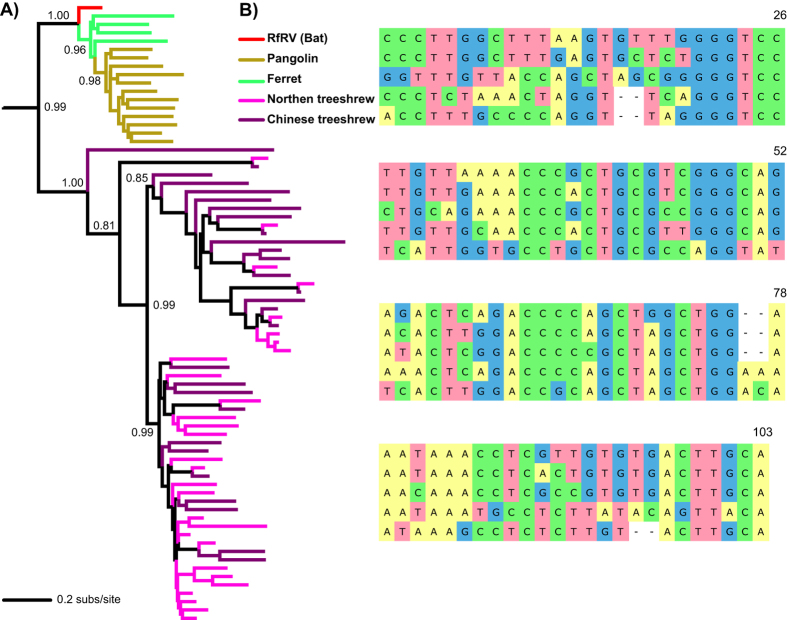
Evolution of RfRV. **(A)** Phylogenetic position of RfRV in gammaretroviruses. Branch lengths are drawn to a scale of amino acid substitutions per site (subs/site). The tree is rooted to human endogenous retrovirus-like element (HERV-E), a Class I ERV (not shown). Bootstrap values higher than 70% are shown. All abbreviations can be found in Table S2. (**B**) Alignment of R (repeat region) of RfRV related LTRs. Detailed information of the tree shrew and mole rat ERVs can be found in Table S3.

To further highlight the ability of bats handling alien ERVs, here we present the unusual finding for the evolution of RfRV (*Rhinolophus ferrumequinum* retrovirus), a retrovirus first discovered in the transcriptome of the greater horseshoe bat^13^. Strikingly, the pan-phylogenomic analysis of Class I ERVs (Fig. S3) reveals that neither its natural host, the greater horseshoe bat, nor the other nine bats, straw-coloured fruit bat, big brown bat, greater false vampire bat, Brandt’s bat, David’s myotis, little brown bat, black flying fox, Parnell’s mustached bat, and large flying fox, that were analyzed, harbor RfRV (or RfRV-like) viruses in their genomes. Instead, pangolin, ferret, northern tree shrew, and Chinese tree shrew have similar viruses integrated in their genomes (Fig. 3A). Phylogenetic reconstruction using Gag and Env proteins support the RfRV position (Fig. S5) observed in the Pol phylogeny (Fig. 3A). Strikingly, the LTR alignment analysis further confirms the relatedness of RfRV with non-bat ERVs from those four host genomes, especially close to the ones harbored by pangolin and ferret (Fig. 3B). Notably, RfRV shares the same two nucleotide deletions and insertions with the viruses from pangolin and ferret (Fig. 3B). The tBLASTn analysis using the Gag, Pol and Env protein sequences as well as LTR mining confirm the above finding with the highest tBLASTn hit sequences representing viruses from those four host genomes. The lack of virus-host congruence (Fig. 3A) as well as the lack of evidence demonstrating RfRV integration in bats suggests that cross-species transmission occurred recently, such that the viruses originated in treeshrews and transmitted via bats to pangolins and ferrets. There is also another possibility that the virus is not fixed in the bat population, similar to koala retroviruses^14^, where RfRV could have endogenized viral forms in some un-sampled bat populations. Notably, the sequenced greater horseshoe bat^15^, which we performed genomic mining, and the host of RfRV^13^ are two continentally separated greater horseshoe bats. Regardless, our data suggests that RfRV (or RfRV-like) viruses originated from non-bat reservoirs and were likely received and spread out by the greater horseshoe bats.

## Conclusion

A previous study reported that rats are probably the major hosts responsible for the diversification of mammalian Class I ERVs^5^. That study included two bat genomes and five rodent genomes. With more rodent and bat genome sequences becoming available we have conducted a new analysis with significantly expanded number of genomes. Our data makes an important discovery that bats are major receiving hosts for ERVs from non-bat origin, and that bats and rodents are equally important as intermediate hosts for ERVs.

Bats and rodents host the majority of zoonotic viruses and have traits such as physiological and ecological factors that are correlated with viral richness in both host groups^16^. Our pan-phylogenomic analyses of Class I and II ERVs highlight that bats and rodents are a major source of origin and transmission of retroviruses to other mammals such as livestock. Due to the close association of bats and rodents with mammalian retroviruses, we propose that both groups combined have significantly contributed to the global spread of retroviruses. Thus, the mechanisms by which mammalian retroviruses are able to readily cross the species barrier to infect bats and rodents would be of interest for future studies.

## Materials and Methods

### Genomic Mining

To mine Class I and II ERVs, a total number of 69 mammalian genomes were downloaded from NCBI Assembly (http://www.ncbi.nlm.nih.gov/assembly/) or Ensembl (www.ensembl.org), consisting of 10 bats, 12 rodents, 8 carnivorans, 6 even-toed ungulates, 5 primates, 4 cetaceans, and others as listed in Table S1. To extract Class I and II ERVs, an *in silico* mining pipeline was constructed using the following procedures: 1) tBLASTn in blastall package^17^ (version 2.2.21), which searched translated nucleotide databases using a protein query, was employed to mine the host genomes using the complete Pol protein sequences of all retroviruses (Table S2), with a cut-off E-value = 1e^−5^ (protein sequences with E-value lower than this value were collected); 2) GeneWise^18^ (version 2.2.0), which compared protein sequences to genomic DNA sequences, was employed to extract the viral protein sequences in the mammalian genomes; 3) complete Pol sequences (consisting of ~880 amino acids for Class I and ~780 amino acids for Class II) were extracted, together with their flanking regions in the genome (10 kb at both ends), which were used for identifying orthologous viral sequences among different hosts; 4) a reciprocal BLASTn method, using a cut-off E-value = 1e^−5^, which searched the queried viral protein sequences against the Reference Sequence (RefSeq) protein database (www.ncbi.nlm.nih.gov/refseq/), was employed. The last method was efficiently used for ruling out false positive hits (i.e. hits from host proteins) extracted during the genomic mining. For example, if one sequence with the lowest E-value hit the host protein first, this sequence was considered a false positive and discarded. Although we aimed to detect Class I (gammaretrovirus-like) and Class II (betaretrovirus-like) ERVs, we used retroviruses of all seven genera as queries which could cover all the endogenous retroviral forms. We then employed a phylogenetic method (see method below) to exclude non-Class I or II ERVs.

### ERVs Extraction

After exclusion of those sequences with large truncated regions and multiple ambiguous amino acids, the remaining sequences were subjected to our developed phylogenetic approach to extract Class I and II ERVs. All sequences were aligned together with 52 exogenous (or endogenous having exogenous forms) retroviruses (Table S2) using MAFFT^19^ (version 7.149). Then the aligned sequences were used to generate a maximum likelihood (ML) tree by employing FastTree^20^ (version 2.1.7). This method was successfully applied for reconstructing the phylogenies of large numbers of ERVs in vertebrate genomes^5^,^21^. The Jones-Taylor-Thornton (JTT) model of amino acid evolution accounting for varying rates of evolution across sites (CAT model, named due to classifying sites into categories) and default setting were used. All Class I, II and III ERVs can be classified into three groups by using phylogenetic inference and Class I ERVs can be rooted to epsilonretroviruses and Class II to alpharetroviruses^3^,^5^,^9^. Based on the above rationale, we were able to extract Class I and II specific ERVs. All sequences having blurred relationships with Class I or II ERVs, for instance epsilonretrovirus-like or alpharetrovirus-like ERVs, were not included in our study.

### Phylogenetic Reconstruction

Class I and II ERVs were collected after data processing and two major phylogenetic trees were generated. To increase the accuracy of ML searching, a “slow” version of computation^20^ using JTT model was employed with the following parameters: -spr 4 (increasing the number of rounds of minimum-evolution subtree-prune-regraft (SPR) moves) and -mlacc 2 -slownni (making the ML nearest-neighbor interchanges (NNIs) more exhaustive). Phylogenetic uncertainty was assessed by the Shimodaira-Hasegawa (SH) test for each split in the tree and was resampled 1,000 times. The Class I phylogenetic ERV tree was rooted to walleye dermal sarcoma virus, an epsilonretrovirus, and Class II to avian leukosis virus, an alpharetrovirus.

### RfRV and RfRV-like Sequence Mining and Phylogenetics

To elucidate the origin of *Rhinolophus ferrumequinum* retrovirus (RfRV), we performed another tBLASTn search using the online genomic mining tool (http://blast.ncbi.nlm.nih.gov/Blast.cgi), in which all available vertebrate genomes (http://www.ncbi.nlm.nih.gov/genome/browse/) were targeted. Strict cut-offs were used in tBLASTn mining: query coverage > 80%; E-value < 1e^−100^; identity > 60%, using concatenated Gag-Pro-Pol and Env sequences of RfRV as two queries, respectively.

The evolutionary history of RfRV and related viruses was inferred using the ML method in PhyML^22^ (version 3.1), including 1,000 bootstrap replications to determine the robustness of specific nodes. The algorithm of Subtree Pruning and Regrafting (SPR) topological moves was used to search the tree space. The ProtTest (version 2.4) was used to select the best-fit model of protein evolution^23^, which were JTT + Γ + F for Gag dataset and WAG + Γ + F for Env.

## Additional Information

**How to cite this article**: Cui, J. *et al.* Bats and Rodents Shape Mammalian Retroviral Phylogeny. *Sci. Rep.* **5**, 16561; doi: 10.1038/srep16561 (2015).

## Supplementary Material

Supplementary Figures

Supplementary Tables
