# Supplementary material for: Bats and Rodents Shape Mammalian Retroviral Phylogeny
Source: Sci Rep. 2015 Nov 9;5:16561. doi: 10.1038/srep16561 (PMC4637884; doi:10.1038/srep16561)
Supplement: Supplementary Figures [file srep16561-s1.pdf]

Supplementary Figure legends

Figure S1. Quantified phylogenetic diversity of ERVs from bats and rodents. The y-axis represents phylogenetic diversity per copy numbers per host species of Class I **(A)** and Class II **(B)**. All the abbreviations can be found in Table S2.

Figure S2. Phylogenetic positions of Class I and II ERVs from host groups other than rodents and bats. (A-D) Trees represent color-highlighted Class I ERVs from even-toed ungulates, carnivorans, cetaceans, and primates; **(E-H)** trees represents Class II ERVs from even-toed ungulates, carnivorans, cetaceans, and primates.

Figure S3. Collapsed phylogenetic Class I ERV tree. Viral branches of rodents are blue highlighted and bats are red. Viruses that form a same-host clade ($n \geq 2$) or a similar-host (i.e. from same host family) clade are collapsed into single lineage for the purpose of visualization only. The abbreviations of the hosts are used to represent viruses; a plus symbol denotes different hosts combined; and abbreviation in a parenthesis represents exogenous virus (or viruses) sharing the same (or similar) host. Branch lengths are drawn to a scale of amino acid substitutions per site (subs/site). The phylogeny is rooted to walleye dermal sarcoma virus, an epsilonretrovirus (marked with a triangle). The SH values (≥0.70) of the collapsed clades (i.e. viruses from same or similar hosts) are not shown for clear visualization only and so of the low SH values (<0.70). Several clades used for transmission pattern analysis are highlighted (with the name of Clade 1.X).

Figure S4. Collapsed phylogenetic Class II ERV tree.

Viral branches of rodents are blue highlighted and bats are red. Viruses that form a same-host clade ($n \geq 2$) or a similar-host (i.e. from same host family) clade are collapsed into

single lineage for the purpose of visualization only. The abbreviations of the hosts are used to represent viruses; a plus symbol denotes different hosts combined; and abbreviation in a parenthesis represents exogenous virus (or viruses) sharing the same (or similar) host. Branch lengths are drawn to a scale of amino acid substitutions per site (subs/site). The phylogeny is rooted to avian leukemia virus, an alpharetrovirus (marked with a triangle). The SH values (≥0.70) of the collapsed clades (i.e. viruses from same or similar hosts) are not shown for clear visualization only and so of the low SH values (<0.70). Several clades used for transmission pattern analysis are highlighted (with the name of Clade 2.X).

Figure S5. Phylogenetic position of RfRV. Phylogenetic Gag (A) and Env (B) trees are constructed using complete protein sequences. XMRV is not included in the Gag dataset and echidna ERV is not included in Env due to truncation of the sequence. One ERV from each host is used and the accession numbers are: for Gag dataset, Map (JPTV01147828.1), Mpf (AEYP01035397.1), Tub (AAPY01444180.1), Tuc (ALAR01208504.1), Nag (AXCS01064927.1), and Heg (AFSB01041127.1); and for Env, Map (JPTV01094840.1), Mpf (AGTQ01013163.1), Tub (AAPY01808430.1), Tuc (ALAR01208504.1), Nag (AXCS01053175.1), and Heg (AHKG01006163.1). Branch lengths are drawn to a scale of amino acid substitutions per site (subs/site). Both trees are rooted to human endogenous retrovirus-like element (HERV-E), a Class I ERV. Bootstrap values higher than 70% are shown. All abbreviations can be found in Table S2.

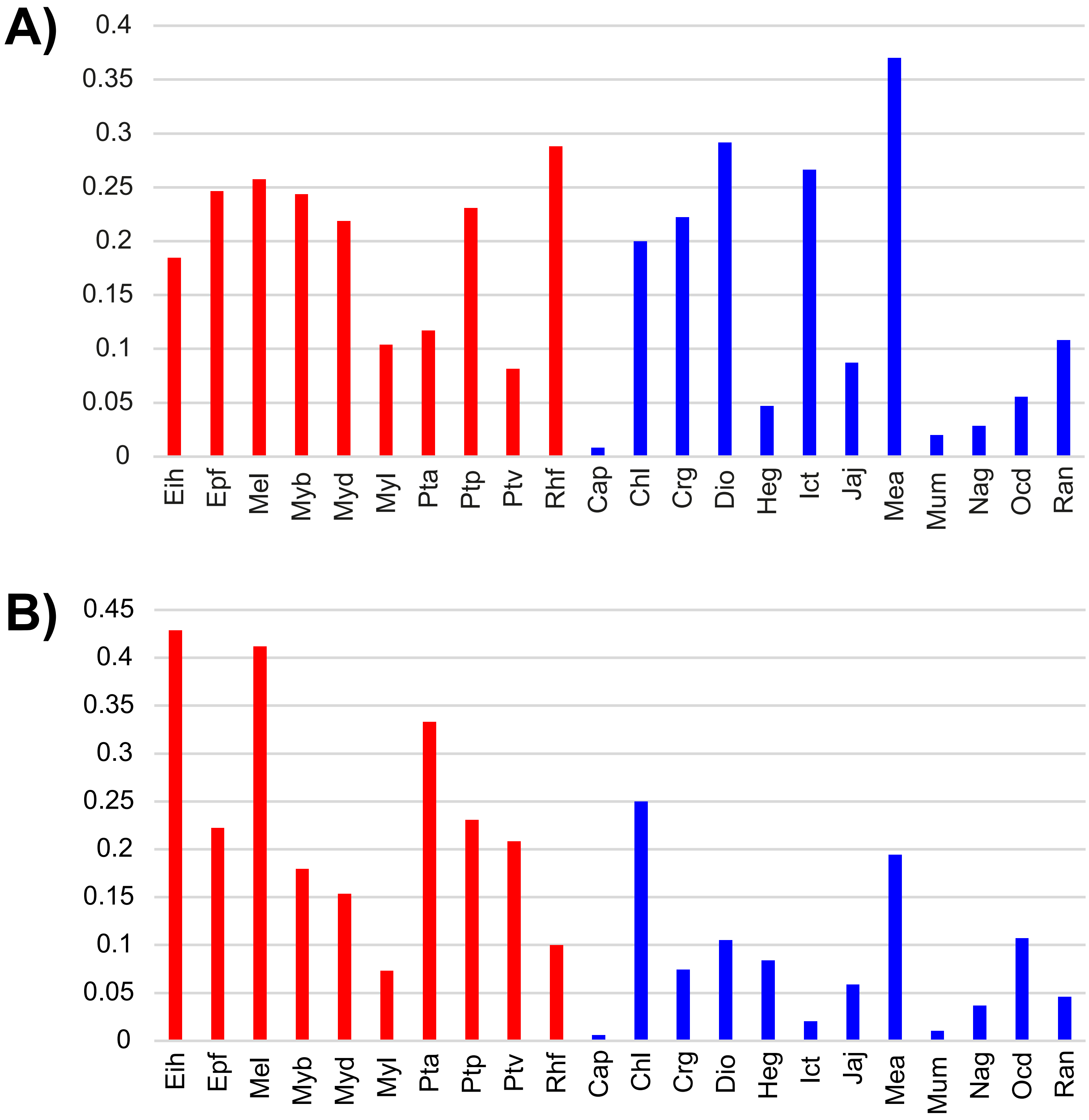
A)
0.4
0.35
0.3
0.25
0.2
0.15
0.1
0.05
0
Eih
Epf
Mel
Myb
Myd
Myl
Pta
Ptp
Ptv
Rhf
Cap
Chl
Crg
Dio
Heg
Ict
Jaj
Mea
Mum
Nag
Ocd
Ran
B)
0.45
0.4
0.35
0.3
0.25
0.2
0.15
0.1
0.05
0
Eih
Epf
Mel
Myb
Myd
Myl
Pta
Ptp
Ptv
Rhf
Cap
Chl
Crg
Dio
Heg
Ict
Jaj
Mea
Mum
Nag
Ocd
Ran

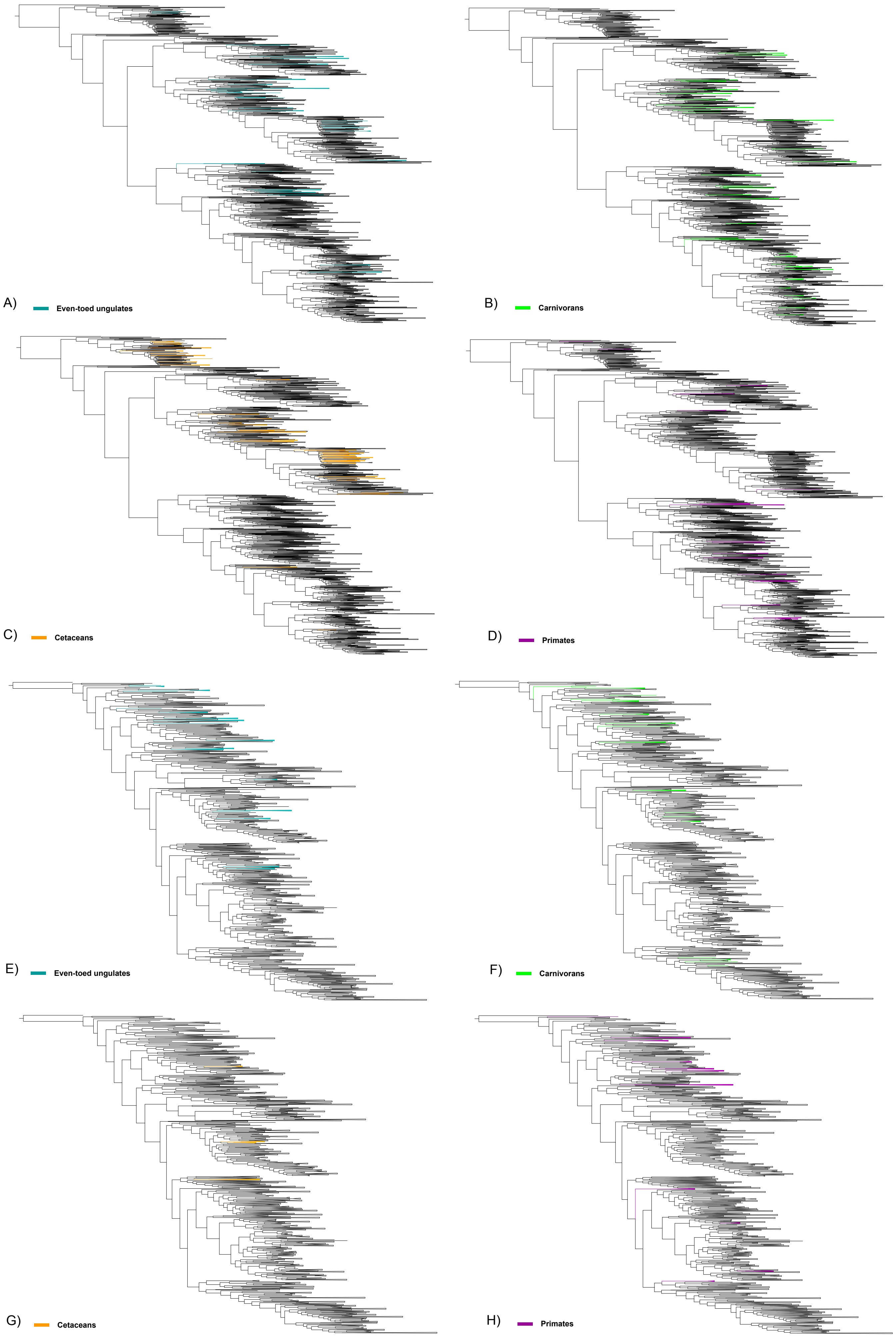
A)
Even-toed ungulates
B)
Carnivorans
C)
Cetaceans
D)
Primates
E)
Even-toed ungulates
F)
Carnivorans
G)
Cetaceans
H)
Primates

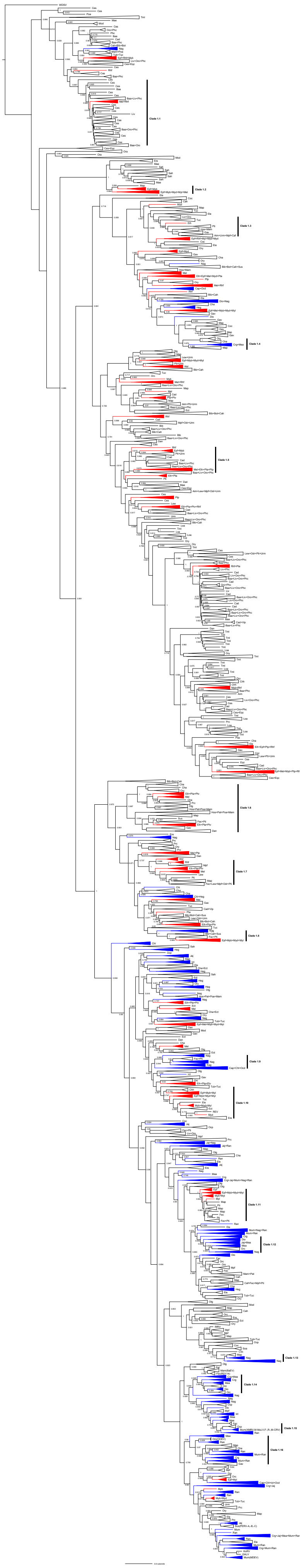

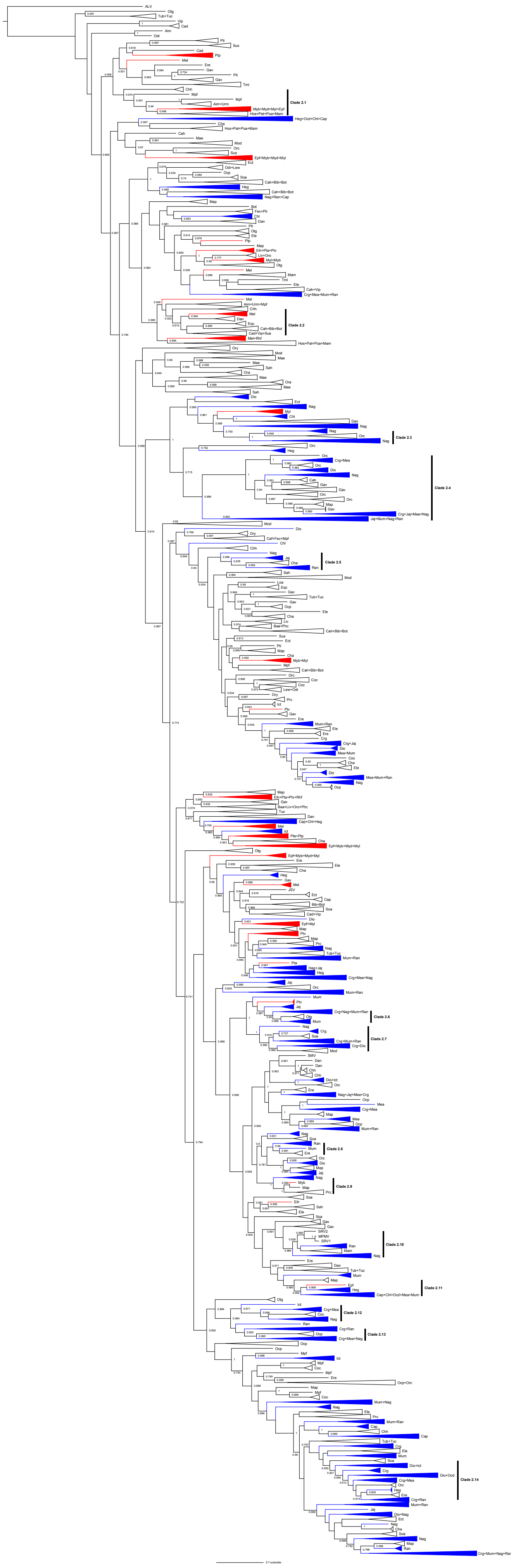

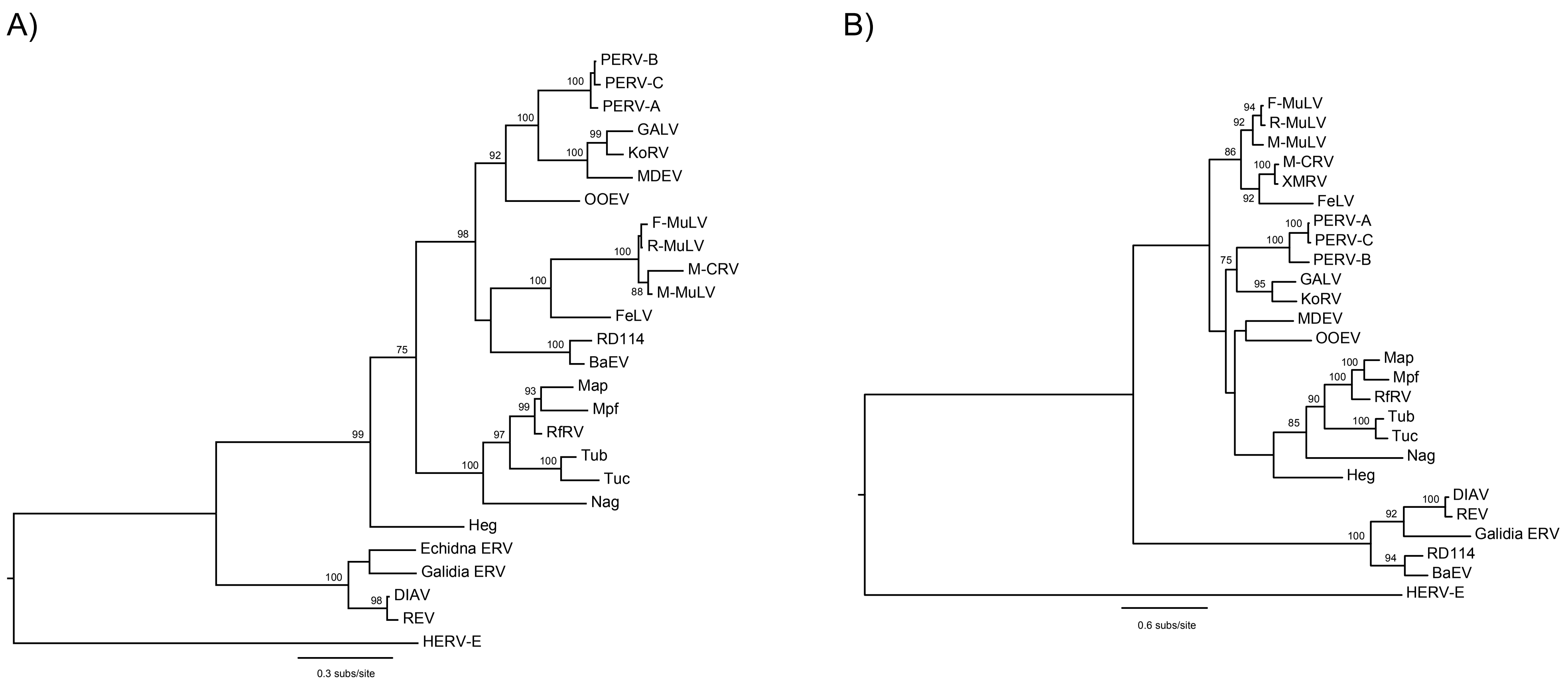
A)
PERV-B
PERV-C
PERV-A
GALV
KoRV
MDEV
OOEV
F-MuLV
R-MuLV
M-CRV
M-MuLV
FeLV
RD114
BaEV
Map
Mpf
RfRV
Tub
Tuc
Nag
Heg
Echidna ERV
Galidia ERV
DIAV
REV
HERV-E
0.3 subs/site
B)
F-MuLV
R-MuLV
M-MuLV
M-CRV
XMRV
FeLV
PERV-A
PERV-C
PERV-B
GALV
KoRV
MDEV
OOEV
Map
Mpf
RfRV
Tub
Tuc
Nag
Heg
DIAV
REV
Galidia ERV
RD114
BaEV
HERV-E
0.6 subs/site
